# Applying a Conservation-Based Approach for Predicting Novel Phosphorylation Sites in Eukaryotes and Evaluating Their Functional Relevance

**DOI:** 10.1021/acs.jproteome.5c00278

**Published:** 2025-07-29

**Authors:** Anton Kalyuzhnyy, Patrick A. Eyers, Claire E. Eyers, Eric W. Deutsch, Andrew R. Jones

**Affiliations:** † Department of Biochemistry, Cell and Systems Biology, Institute of Systems, Molecular and Integrative Biology, 4591 University of Liverpool, Liverpool L69 7BE, U.K.; ‡ Computational Biology Facility, Faculty of Health & Life Science, University of Liverpool, Liverpool L69 7BE, U.K.; § Centre for Proteome Research, Faculty of Health & Life Science, University of Liverpool, Liverpool L69 7BE, U.K.; ∥ 7268 Institute for Systems Biology, Seattle, Washington 98109, United States

**Keywords:** protein phosphorylation, phosphosite, proteomics, proteome, conservation, protein kinase, protein domain, functional annotation, eukaryotic species, model organism

## Abstract

Protein phosphorylation, a key post-translational modification, is central to cellular signaling and disease pathogenesis. The development of high-throughput proteomics pipelines has led to the discovery of large numbers of phosphorylated protein motifs and sites (phosphosites) across many eukaryotic species. However, the majority of phosphosites are reported from human samples, with most species having a few experimentally confirmed or computationally predicted phosphosites. Furthermore, only a small fraction of the characterized human phosphoproteome has an annotated functional role. A common way of predicting functional phosphosites is through conservation-based sequence analysis, but large-scale evolutionary studies are scarce. In this study, we explore the conservation of 20,751 confident human phosphosites across 100 eukaryotic species and investigate the evolution of associated protein domains and kinases. We categorize protein functions based on phosphosite conservation patterns and demonstrate the importance of conservation analysis in identifying organisms suitable as biological models for studying conserved signaling pathways relevant to human biology and disease. Finally, we use human protein sequences as a reference for propagating over 1,000,000 potential phosphosites to other eukaryotes. Our results can improve proteome annotations of several species and help direct research aimed at exploring the evolution and functional relevance of phosphorylation.

## Graphical abstract

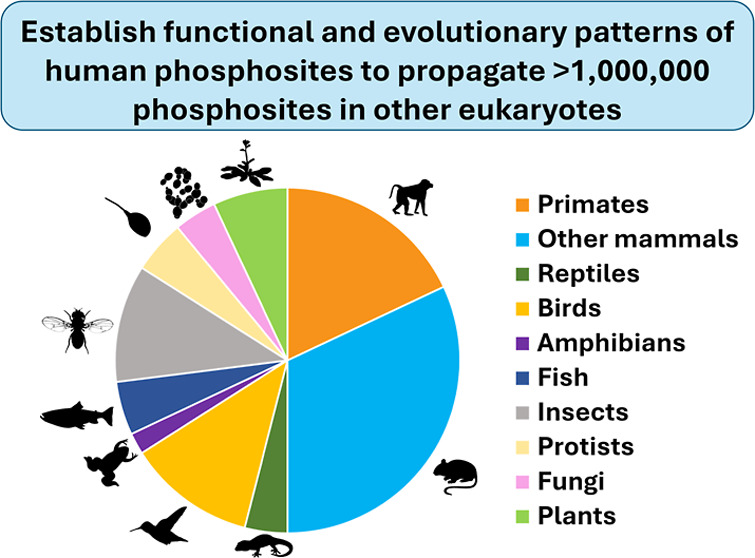

## Introduction

### The Extent of Protein Phosphorylation in Eukaryotes

In proteomics, kinase-regulated protein phosphorylation is perhaps the most important and frequently observed post-translational modification (PTM)^1^ which is well-studied in relation to cell signaling pathways and disease across all life.^2–4^ The extent of protein phosphorylation in various eukaryotic species has been highlighted by several studies that revealed >530 protein kinase-encoding genes in humans,^5–8^ >1000 in *Arabidopsis thaliana*,^9^ 240 in *Drosophila melanogaster*,^10^ and 120 in *Saccharomyces cerevisiae*.^11^ Furthermore, the development and optimization of high-throughput proteomics pipelines such as tandem mass spectrometry (LC–MS/MS) has led to the discovery of large numbers of phosphorylated protein motifs and sites, related primarily to the phosphorylation of canonical (established) serine (Ser), threonine (Thr), and tyrosine (Tyr) residues.^12–17^ Newly identified phosphorylation sites (phosphosites) are characterized and compiled in several publicly available resources, including PhosphoSitePlus (PSP),^18^ PeptideAtlas (PA),^19^ dbPAF,^20^ The Plant PTM Viewer,^21^ and PTMeXchange (https://www.proteomexchange.org/ptmexchange/index.html), all of which accumulate PTM data from eukaryotic species.

However, to our knowledge, most public databases do not account for the phosphosite false discovery rate across large proteomics data sets, which results in the accumulation of false positive identifications.^22,23^ Therefore, the number of “real” phosphosites in those resources may be much lower than reported, and researchers should carefully evaluate the combined evidence for phosphosites before drawing conclusions. Nevertheless, our previous in-depth analysis of phosphorylation data from commonly used resources such as PSP^18^ and PA^19^ revealed that the count of “real” phosphosites in humans alone is likely to exceed 80,000.^22^ We also established a set of over 20,000 “gold standard” phosphosites that had extensive positive identification evidence in both PSP and PA.^22^ Phosphorylation remains an active area of research, with new phosphosites being reported and annotated frequently as proteomics studies multiply.

### Predicting Conservation and Functional Relevance of Phosphosites

Despite numerous phosphosites being reported, only a small fraction of the currently characterized human phosphoproteome has an annotated functional role.^18,24^ This is because the rate of phosphosite discovery is far greater than the rate at which each individual site or motif can be analyzed experimentally. Furthermore, it has been proposed that a significant portion of phosphosites may be “decoy” sites or has no regulatory function at all.^25,26^ The difficulty in distinguishing functionally significant phosphorylated regions from those that do not contribute to protein function is exacerbated by the added complexity of proteins having multiple phosphorylated sites embedded within their sequence, alongside the knowledge that kinases are able to phosphorylate substrates at multiple sites.^27,28^

A common and effective method for predicting functionally significant phosphorylated protein regions is site-conservation analysis. At its simplest, conservation analysis compares the amino acid sequence of a protein in question to the sequences of its homologues and identifies local regions of similarity which may have a common functional implication among the compared proteins.^29^ Conservation analysis of genes and proteins often plays a central role in model organism research, providing a useful approach for investigating human biology and disease.^30,31^ This is highlighted by various studies of organisms such as flies,^32^ worms,^33^ yeast,^34^ and mammals^33,35^ that led to the discovery of fundamental molecular signaling pathways with direct functional connections to human biology.^31,33^

It is hypothesized that functionally significant phosphosites would be highly conserved because their mutations to nonphosphosites would alter protein function and ultimately hinder evolutionary selection.^36,37^ Several studies demonstrated that Ser, Thr, and Tyr phosphosites are indeed significantly more conserved compared to nonphosphosites in general.^24,37^ Our previous profiling analysis of the human phosphoproteome also revealed that phosphosites are on average more conserved than sites with no phosphorylation evidence within the same protein sequences.^22^ Multiple cross-species studies of phosphosites have successfully characterized their conservation and linked it to functions such as cell cycle maintenance and metabolism.^38–40^ However, extensive evolutionary studies that investigate phosphosite conservation across large numbers of species remain scarce.^24,41^ In this study, we provide a deeper insight into general evolutionary and functional trends surrounding human phosphosites by calculating and analyzing phosphosite conservation within specific groups of eukaryotic clades (vertebrates and invertebrates, mammals only, primates only, etc.) and understanding their functional relevance within those groups. We also analyze functional enrichment of human protein sets with different phosphosite conservation patterns using clusterProfiler,^42^ an open-source, user-friendly R package that offers comprehensive analysis and visualization of enriched functions. Additional functional annotations are mapped using the DAVID online tool^43^ which allows us to extract functional terms from bioinformatics databases such as UniProtKB,^44^ KEGG,^45^ and SMART.^46^ Furthermore, we explore phosphosite conservation in relation to protein domains by mapping our data to InterPro,^47^ a powerful resource that integrates domain data from multiple databases. Finally, to further understand the evolution of enzymatic phosphorylation, we analyze general conservation patterns of mapped human Ser/Thr protein kinases.

### Predicting Novel Phosphosites in Eukaryotes

Studies of phosphorylation sites and protein kinases are usually limited to specific species, with the most frequent phosphosite discoveries being made in humans, followed by several model organisms such as mouse, flies, worms, yeasts, and *Arabidopsis*.^18,19^ As a result, the number of reported phosphosites varies between species, with most eukaryotes having little to no evidence of either experimentally confirmed or computationally predicted phosphosites.^18,41,44^ For example, in the January 2023 build of PhosphoSitePlus, there were only 24 characterized eukaryotes with any reported phosphorylation data from low or high-throughput studies.^18^ The process of identifying phosphosites using LC–MS/MS and subsequent proteome annotation can be expensive and time-consuming, and its complexity also depends on the size of the proteome being annotated, as well as the availability of accurate experimental and genomic data for the species from which the proteome originates.^48,49^ As a result, phosphoproteomics studies have not been attempted for most species. In fact, most experiments are designed and funded with the purpose of improving the understanding of vertebrate (notably human) biology and disease, which is often achieved by comparison with a specific set of model organisms that have conserved functions in humans.^30,31,33^

Various computational tools such as ConSurf,^50^ ACES,^51^ Ensembl Compara,^52^ and NetPhos^53^ incorporate algorithms that analyze sequence conservation to predict functionally relevant sites within given protein sequences. Some phosphosite annotations in UniProtKB are also propagated from sequence similarity between a query sequence and a well-annotated homologous sequence with experimentally confirmed phosphosites, provided that the phosphorylated residue and the surrounding motif are conserved in the homologous sequence.^44^ However, the propagations are usually limited between closely related species from the same taxonomic group.^44^ In this study, we expand the scope of eukaryotic phosphosite predictions by mapping conserved “gold standard” phosphosites from the reference human proteome to aligned sites in likely homologous sequences from 100 eukaryotic species.

## Methods

### Establishing Phosphosite Conservation Patterns

To ensure that only the most confident phosphosites were utilized in this study, we exclusively focused on our previously characterized “gold standard” set of phosphosites (i.e., phosphosites which had at least 5 pieces of positive identification evidence from high or low throughput proteomics experiments referenced in PSP and PA databases).^22^ The human data were sourced from pre-existing publicly available data sets, and thus, no additional ethical approval was required.

In total, the analyzed set contained 16,978 Ser, 2747 Thr, and 986 Tyr sites across 5709 proteins (Table S1). For those sites, we calculated conservation percentage scores across 100 eukaryotic species (Table S2) by applying a computational pipeline for evolutionary conservation analysis described in our previous study.^22^ In brief, each human protein sequence with target phosphosites was used as a query in a BLASTp search (BLAST+ 2.10.0 version)^54^ against all 100 eukaryotic proteomes (complete proteomes in the FASTA format are summarized in Supplementary file proteomes.zip at 10.5281/zenodo.15005439). The BLAST output was processed to extract a top significant hit (*E*-value ≤1 × 10^–5^) from each species for each human query. Human sequences were then aligned with their matched hits using MUSCLE (version 3.8.31),^55^ and the resulting multiple sequence alignments are summarized in supplementary file “alignments.zip” (available at 10.5281/zenodo.15005439). From each alignment, percentage conservation scores were calculated for every target human phosphosite and their +1 residue, taking into account any Ser/Thr substitutions in aligned sequences, whereby a site was counted if a Thr in its sequence was aligned with a Ser in the target human sequence and vice versa.

To establish phosphosite evolutionary patterns within eukaryotes, the data were processed to determine percentage conservation scores within specific groups of species including primates (*n* = 18), other mammals (*n* = 32), birds (*n* = 12), fish (*n* = 5), reptiles (*n* = 4), amphibians (*n* = 2), insects/invertebrates (*n* = 11), fungi (*n* = 4), plants (*n* = 7), and protists (*n* = 5). In addition, conservation scores were calculated for broader groupings such as animals (*n* = 84), vertebrates (*n* = 73), and mammals (*n* = 50). To allow protein-level functional analysis, the average conservation of all Ser/Thr and Tyr phosphosites across each described species group was calculated for each protein in the “gold standard” set.

Taxonomic relationships between the selected 100 eukaryotic species were displayed with a phylogenetic tree built with NCBI’s Taxonomy Browser tool^56^ using relevant UniProtKB proteome ID numbers as inputs (Table S2). The resulting phylogenetic tree was annotated and visualized using MEGA (version 10.2.2)^57^ and iTOL (version 5).^58^ Additional silhouette images within the resulting tree were obtained from PhyloPic database (https://www.phylopic.org/).

All proteins in the analysis (*n* = 5709) were then grouped into ten conservation clusters based on similarities in their Ser/Thr and Tyr phosphosite conservation patterns across the described species groups. The clustering was performed automatically using pheatmap package (version 1.0.12) (https://cran.r-project.org/web/packages/pheatmap/index.html) in R programming software, which applied the Euclidean distance method to assess similarity in phosphosite conservation patterns between target proteins and group them into specific clusters. The resulting protein clusters were presented as heatmaps, and each cluster was manually named with an appropriate descriptive label that corresponded to the most observed conservation pattern within the cluster (i.e., at least 50% of proteins within the cluster had to match the label description in terms of their conservation patterns). The assigned labels characterized phosphosite conservation across species groups as “High” (phosphosites are ≥75% conserved) or “Medium” (phosphosites are ≥50% conserved). Similar clustering analysis was performed on individual sites, where target Ser/Thr and Tyr phosphosites were grouped according to their percentage of conservation across the species groups. To highlight the diversity of conservation patterns identified for target phosphosites, multiple sequence alignments for randomly selected proteins that accurately matched the description of the corresponding clusters of interest and had a characterized function in UniProtKB were annotated and visualized in Jalview (version 2.11.2.3).^59^

To compare the conservation of phosphosites likely to be located within ordered or disordered protein regions, the likelihood of finding target sites within those regions was first predicted using metapredict v2.0,^60^ which produced a score from 0 to 1, where a threshold of >0.5 was used for establishing disordered regions. The conservation of Ser, Thr, and Tyr phosphosites was then compared between ordered and disordered protein regions with boxplots created using Tukey’s method,^61^ where each box extended from the first quartile to the third quartile of the data and the whiskers extended from the box to the farthest data point lying within 1.5× the interquartile range from the box.

### Functional Enrichment Analysis of Conservation Clusters

Each protein cluster was analyzed with R package clusterProfiler (version 4.4.1)^42^ to determine the functional enrichment of proteins with certain Ser/Thr and Tyr phosphosite conservation patterns against a control background of all analyzed proteins (*n* = 5709) with at least one phosphosite from the “gold standard” set. For each cluster, a maximum of the 10 most enriched Gene Ontology (GO) terms were selected and visualized as dot plots, where the number of enriched proteins for a given GO term was provided and the statistical significance of the enrichment was measured with adjusted *p*-values. Clusters were counted as enriched for a particular GO term if their Benjamini–Hochberg adjusted *p*-value was <0.1. The functional enrichment analysis of the target protein clusters was extended by utilizing DAVID online tool (version 6.8)^43^ with all analyzed proteins (*n* = 5709) as a control background. For each cluster, the top 10 (where possible) significant (Benjamini–Hochberg adjusted *p*-value <0.1) functional terms with the highest percentage of proteins mapped were identified, with any near synonymous terms being filtered out. In addition to mapping target clusters to GO terms, DAVID analysis was used to determine whether the clusters were enriched for any KEGG pathways,^45^ UniProtKB keywords,^44^ and annotations from domain databases such as SMART^46^ and InterPro.^47^

### Linking Phosphosite Conservation with Protein Domains

All proteins with putative phosphosites were processed via InterProScan (5.66–98.0),^62^ and all protein domains from InterPro^47^ were extracted and cross-referenced with the site-level conservation data using the protein’s UniProtKB ID tag and site position within the protein sequence. In cases in which a single site was mapped to multiple overlapping domain predictions from InterPro, only the first mapped domain term was considered. To identify domains for which phosphosites with specific conservation patterns were enriched, fold enrichment (termed the enrichment factor) was calculated for each domain against a control background of all phosphosites mapped to any InterPro domains. To calculate fold enrichment, a standard equation for enrichment analysis was applied as follows

$$domain\;fold\;enrichment=\frac{a/b}{A/B}$$

*a* = Count of phosphosites with a specific conservation pattern mapped to domain X, *b* = count of all phosphosites with that specific conservation pattern, *A* = count of all phosphosites mapped to domain X, and *B* = count of all phosphosites in the background distribution (i.e., all phosphosites mapped to any protein domain). The domain data were then filtered to only include domains with at least two mapped phosphosites. The top 10 domains with the highest percentage of mapped sites from each Ser/Thr and Tyr conservation cluster were visualized, and log_2_ (fold enrichment) was presented for easier interpretation.

### Conservation Analysis of Associated Protein Kinases

All kinase-substrate mappings for our target Ser/Thr phosphosites were obtained from a published atlas of substrate specificities for the majority of the human kinome which computationally ranks kinases in terms of their relative likelihood of phosphorylating a motif with reported human phosphosites.^27^ For each of the analyzed phosphosites, we selected the top ranked kinase match from the atlas. Several kinases were selected per site if they had the same maximum likelihood score.

To investigate conservation patterns of the matched kinases, we first identified their established orthologue groups by searching UniProtKB^44^ and extracting group identification numbers for the OrthoDB database (v11)^63^ which provides high-quality orthologue annotations. Each mapped OrthoDB group was then processed to extract its complete list of unique species containing protein orthologues of the target kinases. To ensure consistency in terms of species selection relative to the phosphosite conservation analysis, the list of species per OrthoDB group was cross-referenced with our selection of 100 eukaryotes (Table S2) to ultimately determine the extent of kinase conservation. The kinases were clustered according to their conservation patterns across groups of eukaryotes by using a similar method applied for phosphosite conservation analysis. Finally, the conservation scores of Ser/Thr phosphosites and their top matched kinases in the selected 100 eukaryotic species were compared using linear regression and assessed with the *R*-squared (*R* ^2^) coefficient.

### Conservation of Phosphosites Involved in Protein–Protein Interactions

All phosphosites from the “gold standard” set and their conservation scores were merged with data from the PTMint database,^64^ which contains curated lists of phosphosites that have been shown to influence (“enhance” or “inhibit”) protein–protein interactions via several different experimental techniques (file “PTM experimental evidence.csv” at https://ptmint.sjtu.edu.cn/Download; June 2025). We classified our phosphosites as having evidence of involvement in protein–protein interactions if a site was recorded in PTMint (combining “enhance” and “inhibit” categories to give higher statistical power) and no evidence if not. We compared conservation across 100 species of Ser, Thr, and Tyr phosphosites involved in protein–protein interactions to those that were not with boxplots using Tukey’s method^61^ and calculated per residue *t* tests to look for significant differences.

### Predicting Phosphosites across Eukaryotes

Multiple sequence alignments between target human proteins and their potential homologues from selected species groups were processed to identify all amino acids in the matched sequences which were aligned with target Ser, Thr, and Tyr phosphosites in the human sequences. In addition, we analyzed proximal amino acids adjacent to the aligned sites at the +1 position (C-terminal to the site of phosphorylation) which are often involved in facilitating substrate recognition by proline-directed kinases and are well-characterized within common phosphorylation motifs.^13,65,66^ For every species in each alignment, if both, the amino acid that is aligned with the human phosphosite and its +1 adjacent site, were conserved in the human sequence (considering Ser/Thr substitutions), then that amino acid was predicted to be a phosphosite. To validate the resulting phosphosite predictions, phosphorylation data were also extracted for mouse and *Arabidopsis* sequences from PSP^18^ and Plant PTM Viewer^21^ databases, respectively, in order to determine how many of the predicted phosphosites in those species had experimental phosphorylation evidence. Additional validation of our predictions was performed by assessing the likelihood of a given mouse site having any experimental phosphorylation evidence and also being supported by strong evidence (>5 pieces of evidence in PSP). The resulting phosphosite predictions across 100 eukaryotic species were summarized into an easily accessible file that can be used to support the annotations of multiple eukaryotic proteomes.

## Results and Discussion

### Evolutionary and Functional Analysis of Human Phosphorylation Sites

In this study, we evaluated the conservation of human phosphosites across 100 eukaryotic species ranging from primates and other vertebrates to plants and fungi. By analyzing average Ser/Thr and Tyr phosphosite conservation from each target human protein, we split the phosphoproteins into independent clusters according to their similarity of phosphosite conservation patterns within specific groups of eukaryotes. Overall, our analysis successfully identified distinct phosphosite conservation patterns of human proteins (Table 1, Figure 1 and Table S1).

**Table 1:**
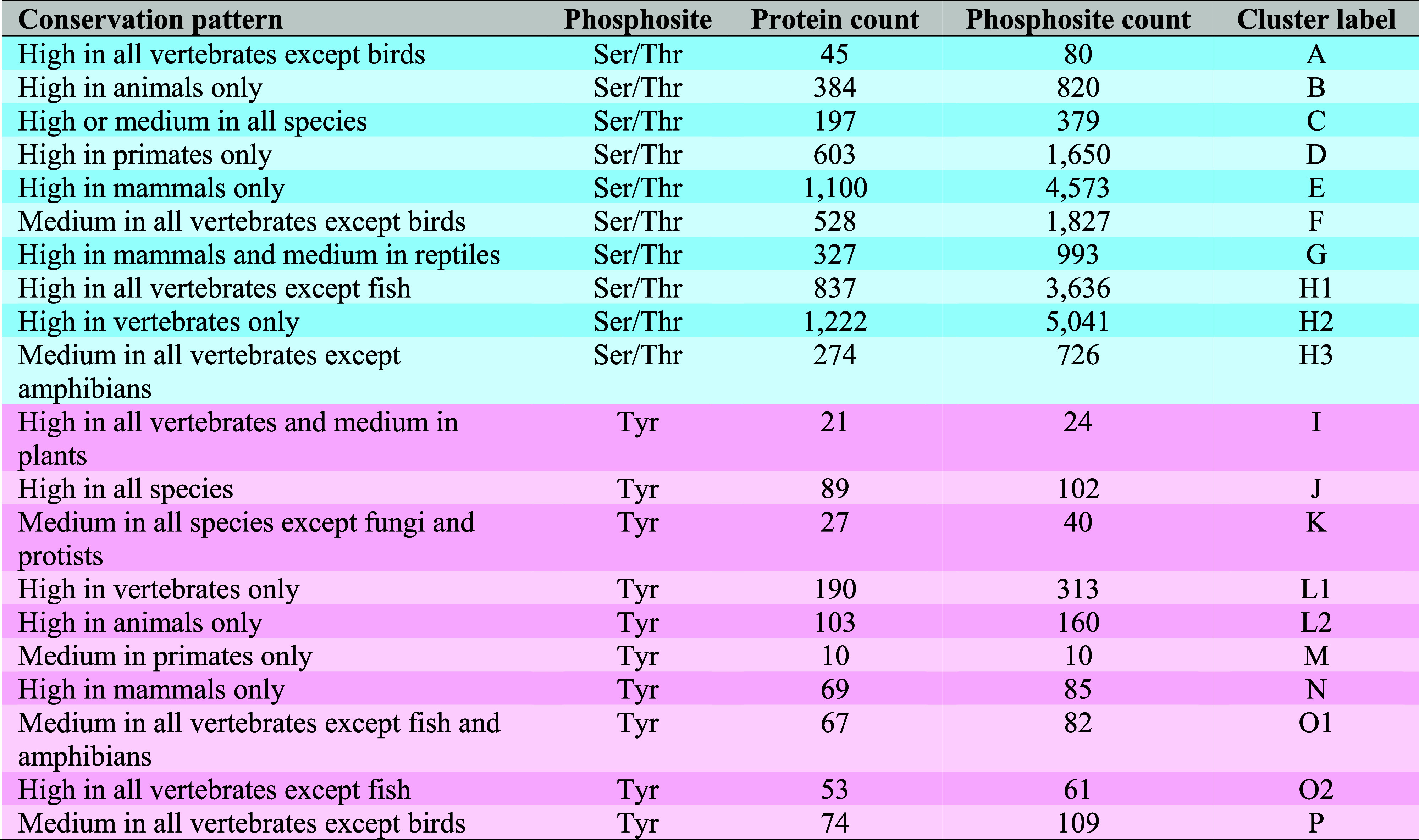
Independent Clusters of Human Phosphoproteins Split According to Their Similarity in Average Ser/Thr and Tyr Phosphosite Conservation Patterns across Established Species Groups^a^

a High and medium conservation indicates that phosphosites are ≥75% and ≥50% conserved across the species in each cluster, respectively.

**Figure 1:**
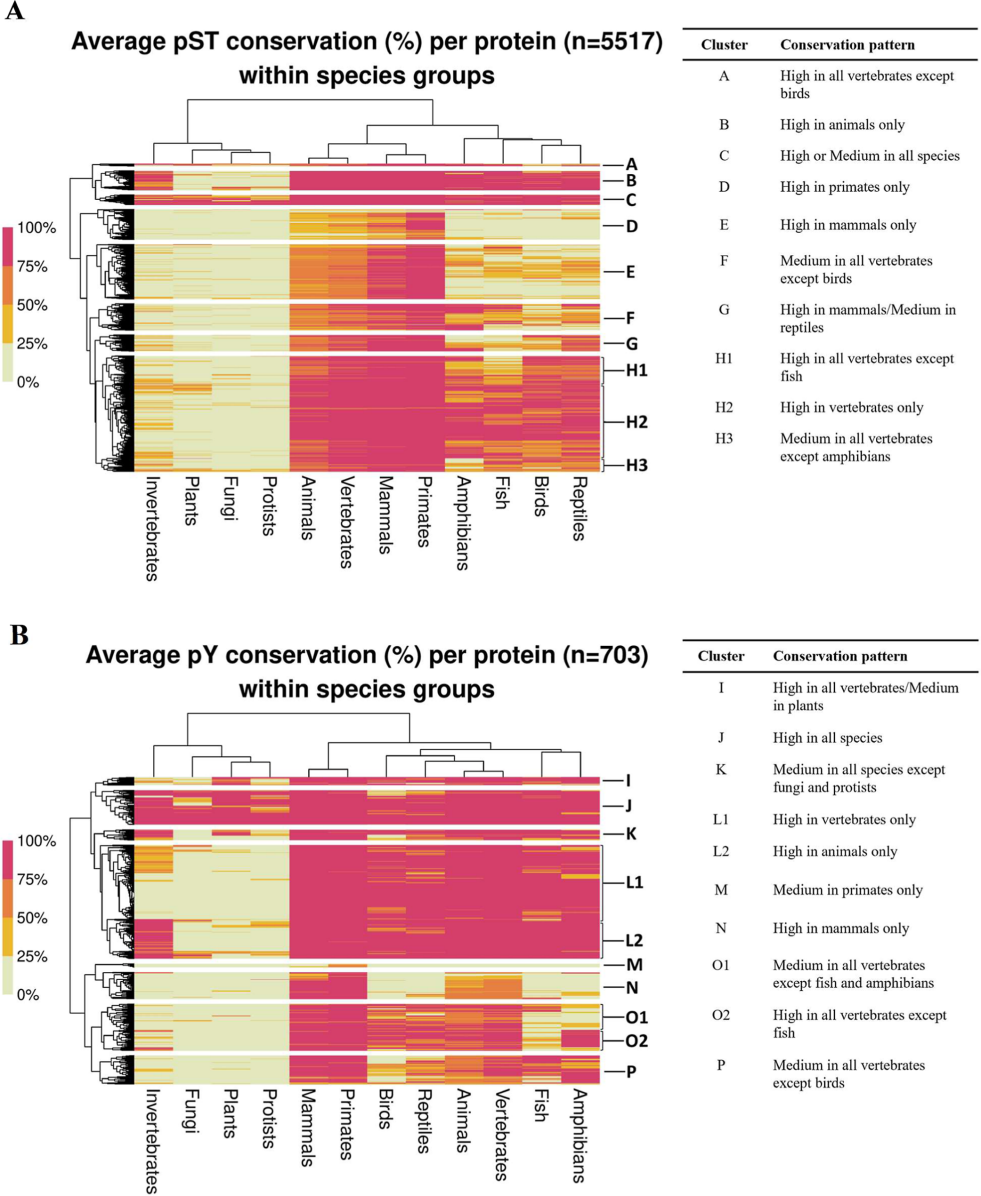
Conservation patterns of (A) Ser/Thr and (B) Tyr phosphosites from human proteins across the groups of eukaryotic species. Each row in the heatmap represents an individual human protein and its phosphosite conservation across specific species groups which are separated into columns. Conservation is scored as a percentage out of all species per group and reflected by a color gradient divided at quarterly intervals. The proteins are clustered based on their similarity in conservation patterns using the Euclidean distance method. For each cluster, a label is assigned which describes the most observed conservation pattern (i.e., at least 50% of proteins in the cluster follows the described phosphosite conservation pattern), where high and medium conservation refers to conservation scores of ≥75% and ≥50%, respectively. The total number of analyzed proteins containing target phosphosites is given by *n*.

The diversity of the established conservation patterns was further highlighted by multiple sequence alignments of individual human protein examples (Figure 2) (all complete alignments can be found in supplementary file “alignments.zip”, available at 10.5281/zenodo.15005439).

**Figure 2:**
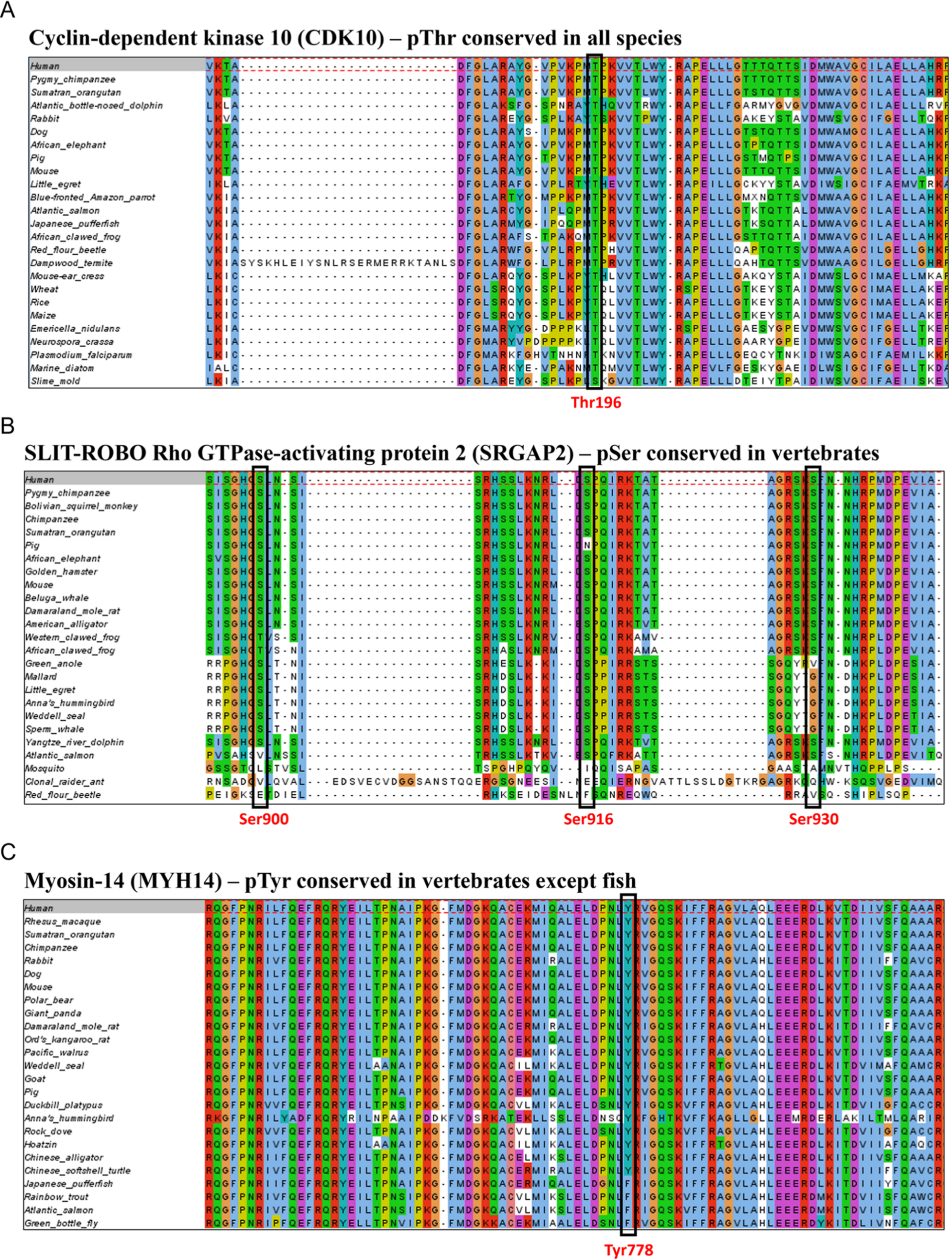
Sections of multiple sequence alignments of target human proteins with different conservation patterns of phosphosites across eukaryotic species. For each alignment, the sequence of the human protein containing the phosphosites is located at the top and the aligned protein sequences of potential orthologues from other species are given below. Phosphorylated sites are marked by black rectangle boxes and their location within the human sequence is provided underneath in red. Alignments were annotated in Jalview.

The differences in conservation patterns of human phosphosites were likely a result of their functional relevance within groups of species in which they were conserved.^24,36,37^ To investigate this hypothesis further, we performed a functional enrichment analysis of proteins with different phosphosite conservation patterns to identify conserved functions that are potentially regulated by those phosphosites. Indeed, we found that some phosphosites were involved in ancient protein functions relevant to all eukaryotic life forms, such as regulation of cell cycle and metabolism, while others were likely contributing to relatively novel functions, such as brain and muscle development, cell motility, and immune response, found in species more closely related to humans (notably mammals or primates) (Figures 3 and S1).

**Figure 3:**
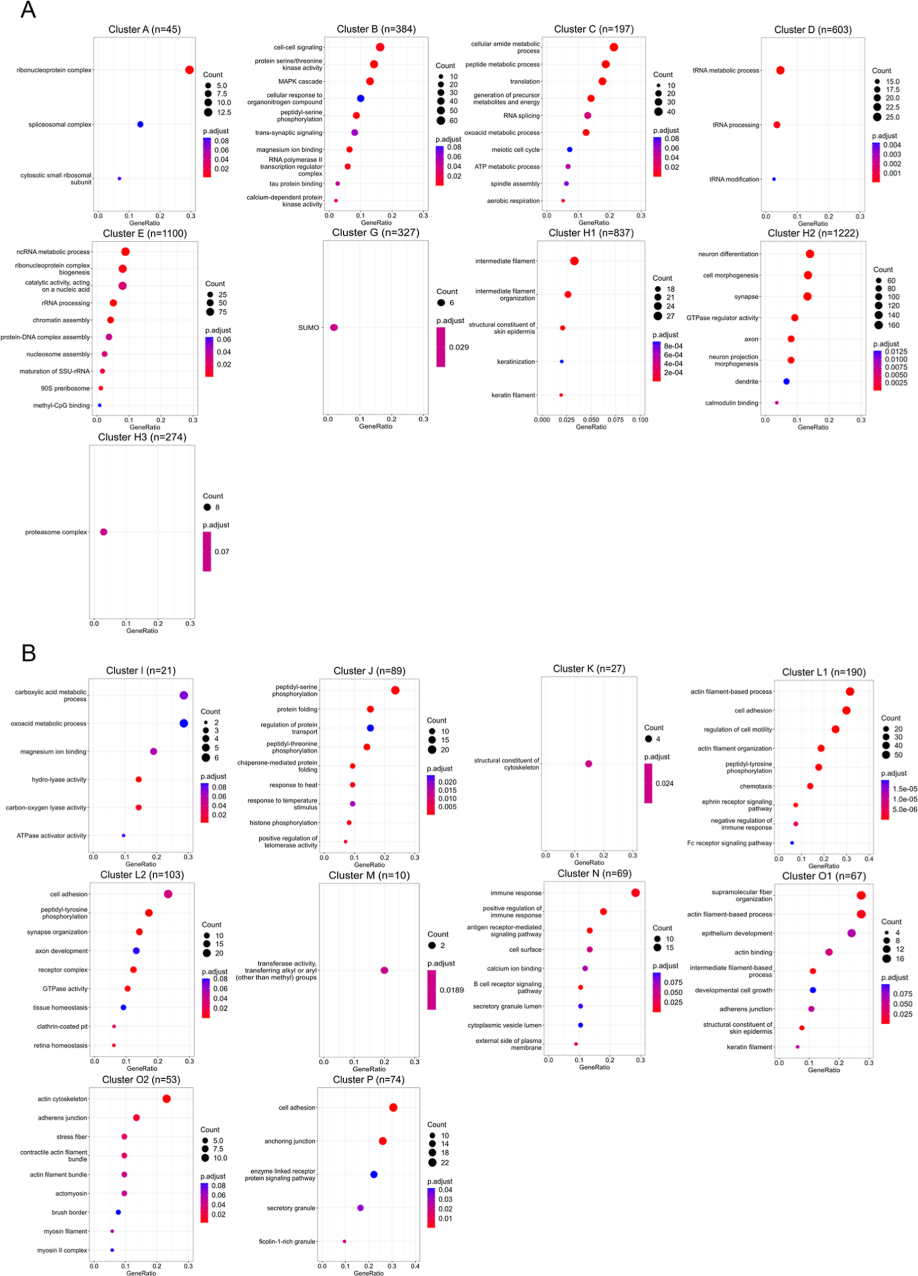
Functional enrichment for GO terms of human phosphoproteins with different (A) Ser/Thr and (B) Tyr phosphosite conservation patterns (please refer to Table 1 or Figure 1 for conservation cluster labels). The enrichment is visualized with dot plots generated using clusterProfiler. In each dot plot, the dots represent protein sets enriched for a specific functional term described on the *y*-axis. The size of the dots reflects the number of proteins in the enriched set, and the color corresponds to the significance of a functional enrichment determined by the Benjamini–Hochberg adjusted *p*-value. The position of the dots on the *x*-axis indicates the proportion of enriched proteins out of all analyzed proteins in the protein set (GeneRatio). The total number of proteins in each set is given by *n*.

We linked the conservation patterns of mapped phosphosites with their associated domains to further understand their evolution and functional relevance in eukaryotes. By identifying frequently observed (enriched) domains per conservation cluster (Figure 4), we provided further evidence to support our results from the functional enrichment analysis.

**Figure 4:**
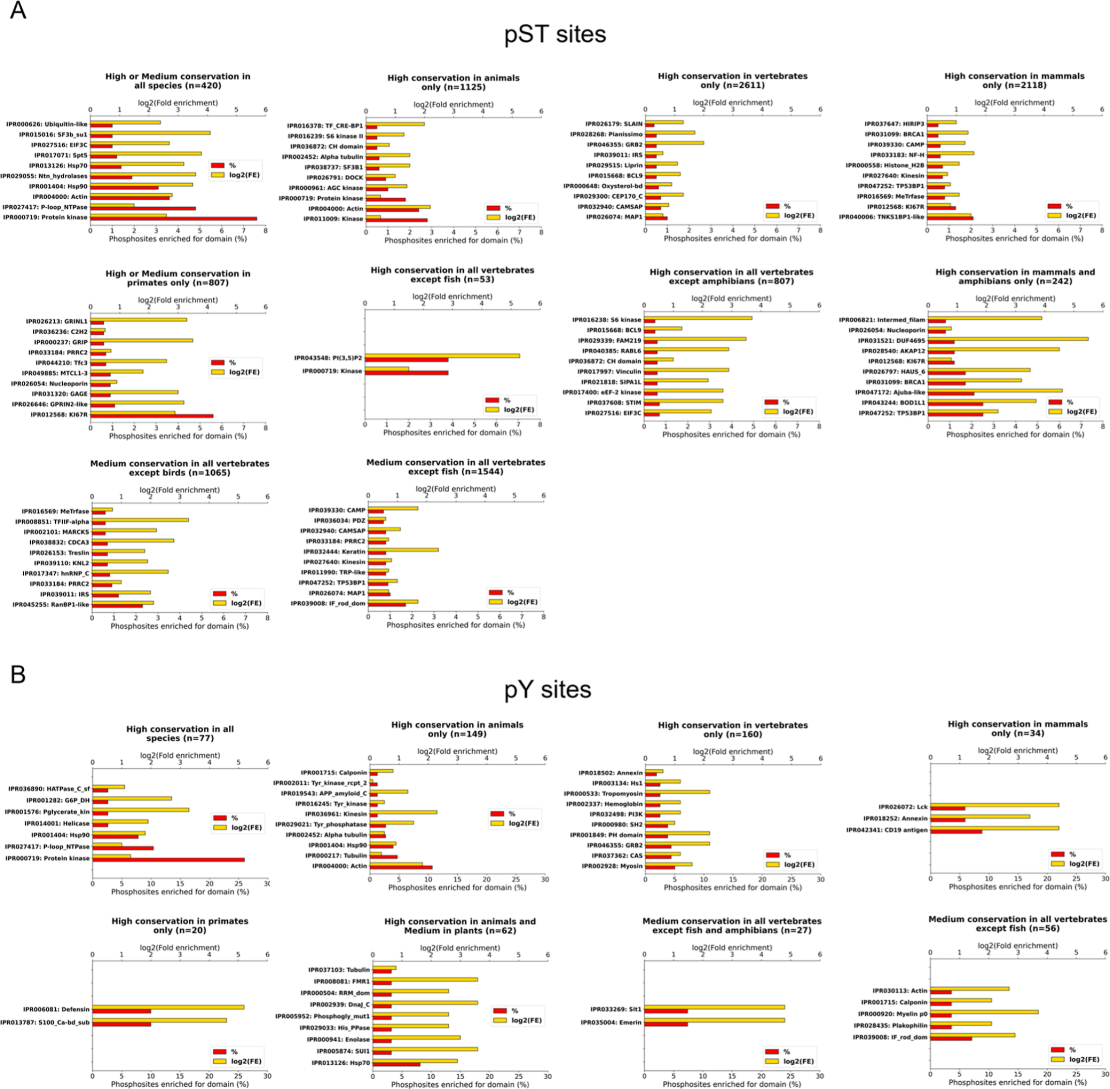
Protein domains from InterPro database (*y*-axis) for which (A) Ser/Thr and (B) Tyr phosphosites with specific conservation patterns across eukaryotes were enriched against a control background of all phosphosites mapped to any InterPro domains. For each conservation pattern, the percentage (%) of phosphosites with that pattern mapped to a specific domain is given, as well as the log_2_ (fold enrichment). The total number of phosphosites with a particular conservation pattern mapped to any InterPro domain ID is presented by *n*.

First, we identified 197 and 89 proteins in which Ser/Thr and Tyr phosphosites, respectively, were on average highly conserved in all species, ranging from mammals to plants and single-celled organisms (Figure 1A, cluster C; Figure 1B, cluster J). We linked those proteins to ancient functions relevant across all life such as translation, metabolism, cell cycle regulation, and the thermal stress response (Figure 3A, cluster C; Figure 3B, cluster J). For example, Thr196 site of the human protein Q15131 (cyclin-dependent kinase 10; CDK10) was well-conserved across all species (Figure 2A) and its phosphorylation plays a key role in regulating cell proliferation and transcription regulation.^67^ This provides further evidence for the functional significance and conservation of CDKs across life, with many CDKs having been well-characterized across multiple species.^68^ Additional DAVID analysis of proteins with phosphosites conserved in all species (Figure S1A, cluster C; Figure S1B, cluster J) revealed enrichment for term “acetylation” which suggests that this PTM, along with phosphorylation, plays a role in key biological functions across all life, potentially as part of PTM crosstalk.^69^ Finally, phosphosites conserved across most species were linked to likely ancient protein domains found in kinases, heat shock proteins, and actin proteins (Figure 4). However, we do not rule out experimental biases meaning that different PTM sites might have been more heavily studied in conserved proteins involved in, for example, the cell cycle.

We also found numerous instances where human Ser/Thr phosphosites were conserved in broader species groups such as animals, vertebrates, or mammals (Figure 1A and Table 1). For example, Ser900, Ser916, and Ser930 sites in human protein O75044 (SLIT-ROBO Rho GTPase-activating protein 2; SRGAP2) were conserved in most vertebrates but absent in insects (Figure 2B). It is likely that aligned insect sequences are not true orthologues of the human proteins as indicated by the lack of any conserved motifs in the analyzed phosphorylated region (Figure 2B). There were also no significant matches in BLAST (*E*-value ≤1 × 10^–5^) for human SRGAP2 in plants, fungi, and protists. This conservation pattern can be rationalized by a functional relevance of SRGAP2 in neuronal morphogenesis during the development of the cerebral cortex associated with higher brain functions, which are expectedly absent in insects, plants, fungi, and protists.^70,71^ This connection was further highlighted by a functional enrichment analysis, which revealed a general enrichment of associated proteins for brain development-related terms such as “neuron differentiation”, “synapse”, and “axon” (Figure 3A, cluster H2). Most importantly, the conservation of the identified pathways regulated by proteins with Ser/Thr phosphosites suggests that the selected vertebrates can be confidently employed as model systems for studying human brain development.

Interestingly, we observed that 613 proteins (11%) contained phosphosites that were only conserved in primates, indicating their potential functional relevance in relatively novel functions which diverged from other animals (Table 1; Figure 1A, cluster D; Figure 1B, cluster M). A functional enrichment analysis of those proteins inferred their involvement in tRNA processing and association with zinc finger-related terms including Kruppel-associated box (KRAB) zinc finger proteins (Figures 3A and S1A, cluster D). In fact, previous studies also characterized groups of KRAB proteins which rapidly evolved in primates and adapted to regulate complex pathways involved in brain development.^72,73^ Furthermore, some proteins were characterized as G-antigen (GAGE) proteins (Figure S1A, cluster D). Those proteins have been known to play a regulatory role in primate germ cell development and were proposed as candidates for immunotherapy of cancer due to their expression in many cancer tissues.^74^ The analysis of protein domains also linked Ser/Thr phosphosites conserved in primates to the “GAGE” domain (Figure 4A). Additionally, we found strong enrichment of Ser/Thr phosphosites conserved in primates and mammals for the “KI67R” domain which is associated with genome stability and mammalian immune response against highly proliferative cells.^75^ Phosphosites associated with this domain were primarily conserved in primates, suggesting its potential involvement in additional, primate-exclusive signaling pathways (Figure 4A). A similar pattern was found for Tyr phosphosites conserved in mammals or primates and enriched for protein domains known to be involved in immune response such as “Lck”,^76^ “Annexin”,^77^ and “Defensin”,^78^ further emphasizing that phosphorylation plays a central role in immune system pathways of higher eukaryotes (Figure 4B). These results highlight that identifying human phosphosites that are conserved in closely related species can extend the availability of genetically similar species for studying human neuronal and immune systems, as well as supporting the development of preclinical models for drug development.

Another noteworthy conservation pattern was established for 890 (16%) proteins in which phosphosites were conserved in most vertebrates except fish (Figure 1A, cluster H1; Figure 1B, cluster O2). Functional analysis of those proteins revealed strong enrichment for terms associated with keratin and myosin (Figure 3A and Figure S1A, cluster H1; Figure 3B and Figure S1B, cluster O2). Moreover, the domain analysis of phosphosites with medium conservation in all vertebrates except fish revealed enrichment for the “IF_rod_dom” domain (Figure 4) involved in the formation of intermediate filaments.^79,80^ First, this suggests that the conserved phosphosites may be involved in the formation of intermediate filaments during keratinization, a process required for the development of epidermis.^81^ It is likely that those signaling pathways are absent in fish because they are not necessary for the survival in an aquatic environment. This conservation pattern was further highlighted by evolutionary studies which similarly identified groups of keratin proteins only conserved in tetrapods (i.e., four-legged vertebrates which evolved later than fish) and linked those proteins to biological pathways involved in the development of tissues and organs such as skin, hair, and nails necessary for protection against the friction caused by terrestrial movement.^82,83^ Second, the enrichment for myosin indicates the involvement of the associated proteins and their phosphosites in cell signaling and motility pathways likely absent in fish. One such protein is Q7Z406 (Myosin-14; MYH14), in which the human Tyr778 was conserved across most vertebrates but was mutated to phenylalanine in fish (Figure 2C). This evolutionary pattern was also confirmed in another study which identified several myosin domains that emerged as a result of divergent evolution between fish and tetrapods.^84^ This example highlights a functional relevance of tyrosine phosphorylation in tetrapods and helps understand the evolution of established myosin-related functions such as cell signaling and motility.^85,86^ In addition, MYH14 has been linked to hearing loss in humans^87^ and the species in which the functional Tyr phosphosite was conserved can be used as potential clinical models to further investigate human disease.

We were unable to link some of our clusters (including clusters F, G, H3, K, and M) to specific biological functions that might have helped further clarify the observed conservation patterns. This is likely a result of a small sample size of proteins involved in those clusters or weaker enrichment, which did not yield any conclusive results. It is also possible that there were sequencing or annotation errors in proteomes of certain species used in our analysis or within the specific protein sequences aligned with our human targets. This could in turn have led to errors in multiple sequence alignments, causing inaccuracies in the characterization of phosphosite conservation patterns and functional enrichment predictions as demonstrated by several studies.^88–90^ Nevertheless, we were able to showcase the relevance of conservation analysis in inferring a range of different protein functions likely regulated by phosphorylation and selecting species that can be used as genetic models to support human studies.

### Linking Phosphosite Conservation to Protein Domains and Regions

In total, we successfully mapped 11,531 out of 20,751 (56%) of our analyzed Ser, Thr, and Tyr phosphosites to 4504 different InterPro domain identifiers (Table S1). It is likely that the unmapped phosphosites had an independent functional role outside of any established protein domain or belonged to a recently discovered protein which has not yet been covered in InterPro. Indeed, we observed that the vast majority (83%) of the target phosphosites were found in disordered protein regions (Table S1; Figure S3), supporting previous structural evaluations investigating phosphosite localization.^91–93^ These observations can be broadly explained by disordered regions being more readily accessible as substrates for catalysis, alongside the need for many functional phosphosites to regulate protein function through surface-based recognition mechanisms.

We observed that phosphosites in ordered protein regions were generally more conserved than those in disordered regions (Figure S3), likely because they are part of phosphorylation hotspots or protein domains that regulate ancient eukaryotic functions.^24^ Interestingly, we also found phosphosites within domains of unknown function (DUFs) (Table S1 and Figure 4A) which are described as families of uncharacterized proteins that do not currently have a known function.^94^ This suggests that phosphorylation could play an important role within the associated DUFs and that our results, along with functional profiling, can ultimately be used to improve their annotation.

### Conservation of Human Kinases

Out of 19,765 Ser and Thr sites from the “gold standard” set, we mapped 18,752 (95%) sites to 301 kinase candidates from the kinome atlas^27^ (Table S1). Out of the mapped sites, 543 (3%) sites had multiple kinase candidates with the same maximum match probability score in the atlas (Table S1). To discover the evolutionary relationships among the mapped kinases, we linked them to the data in the OrthoDB database and identified 101 unique orthologue groups (Table S3). On average, in the mapped OrthoDB orthologue groups, there were 2964 orthologues (range: 97–8739 proteins per group) from 709 different species (range: 83–1238 species per group) (Table S3). To ensure consistency with phosphosite data analysis in terms of species selection and conservation pattern clustering, we only investigated kinase conservation out of the 100 analyzed eukaryotes, whereby a kinase was considered conserved in our selected species if those species were found in the OrthoDB group mapped to that kinase. In total, we found that 92 of our selected 100 species were also present in the OrthoDB analysis (Table S1), which allowed us to perform an analogous conservation clustering analysis for the kinases.

In the initial clustering analysis, we found that the conservation signal was weak for birds, likely because the specific bird species selected in our analysis have not been extensively studied in terms of kinase substrate specificity or have poor proteome annotations. Having excluded birds from the analysis, we obtained much clearer and more interpretable kinase conservation results (Figure S4). Similarly, the conservation signal was generally weak for the protist group, which was likely because two out of five analyzed protists, *D. discoideum* and *T. pseudonana* were not found in any of the mapped OrthoDB groups (Table S1). Nevertheless, we identified several interesting conservation patterns for the target human kinases, including those conserved across all species and those found exclusively in vertebrates or in the broader group of higher eukaryotes (Figure S4).

For example, several members of the human G protein-coupled receptor kinase (GRK) family had orthologues in other animals, suggesting their involvement in regulating signal transduction pathways associated with animal evolution (Figure S4). Previous evolutionary analysis of GRK enzymes concluded that they likely evolved in animals to allow appropriate control of rapid signaling responses.^95^ In addition, we found multiple human kinases that were conserved across most eukaryotic species, indicating their likely involvement in regulating ancient protein functions through phosphorylation (Figure S4). For example, members of CDK, p21-activated kinase (PAK), and mitogen activated protein kinase families regulate proliferative pathways that are conserved across eukaryotic life.^96–98^ Our results also highlight the general diversity of kinase conservation across eukaryotes.

To further explore the evolution of protein phosphorylation, we attempted to compare the conservation patterns between kinases and their associated phosphosites but did not find any strong associations, as indicated by R^2^ coefficient of 8 × 10^–5^ (Figure S5). For example, when analyzing kinases which were conserved in most of our eukaryotes (Figure S4, cluster C4), we discovered that several of their associated phosphosites were also strongly conserved. Conversely, many human phosphosites exhibited weak conservation (for example, only conserved in primates) but were phosphorylated by ancient CGMC family kinases such as CDKs or MAPKs (Table S1).

It is possible that the lack of strong conservation signal between phosphosites and their kinases could be due to how the kinases were mapped, with our analysis critically depending on the accuracy of the kinase mappings in the kinome atlas.^27^ For this study, we selected the top scoring kinase hits from the kinome atlas for the target phosphosites. However, it is well-established that multiple kinases can phosphorylate the same phosphosite and conversely that a single kinase can phosphorylate multiple sites. In addition, our methods for estimating orthologous relationships between kinases and their target phosphoproteins were fundamentally different. As such, any individual associations (or lack thereof) between the conservation patterns of phosphosites and their associated kinases based on the current data may not be definitive and may not fully grasp the complexity of kinase-mediated phosphorylation networks. Nevertheless, we provide a comprehensive starting point for follow-up investigations of the coevolution between kinases and their protein substrates in eukaryotes.

### Conservation of Phosphosites Involved in Protein–Protein Interactions

To determine the conservation of phosphosites involved in regulating protein–protein interactions, we merged our phosphosites from the “gold-standard” set and their conservation with PTMint data. We found that both Ser and Thr phosphosites with the known roles in protein–protein interactions showed significantly higher (*p* < 0.05) site conservation compared to sites with no evidence of involvement, indicating that these are sites of high functional importance across taxa (Figure S6).

Interestingly, Tyr phosphosites showed no significant difference in conservation (*p* > 0.05). However, Tyr phosphosites in general are more highly conserved than Ser/Thr phosphosites, so we might tentatively conclude that Tyr phosphosites are generally highly functionally significant, whereas a proportion of Ser/Thr phosphosites have lower functional significance, which can be detected by this analysis.

### Predicting Phosphorylation Sites in Eukaryotes

We used high-quality human phosphosites (Table S1) as a reference set to infer phosphorylation in other eukaryotes by studying multiple sequence alignments between target human protein sequences and aligned sequences from selected eukaryotes. In total, we predicted >830,000 Ser, >140,000 Thr, and >56,000 Tyr potential phosphosites in the analyzed species (Figure 5 and Table S4). The majority of human phosphosites can be propagated to primates, likely due to their close evolutionary relationship with humans, leading to similarities in protein sequences and their common functional relevance. As expected, with increasing evolutionary distance from humans, the proportion of conserved sites generally decreased (Figure 5). Nevertheless, we predicted hundreds of potential phosphosites in lower eukaryotes such as plants, fungi, and protists based on their sequence alignment with human sites and likely involvement in functions regulated by phosphorylation (Figure 5 and Table S4).

**Figure 5:**
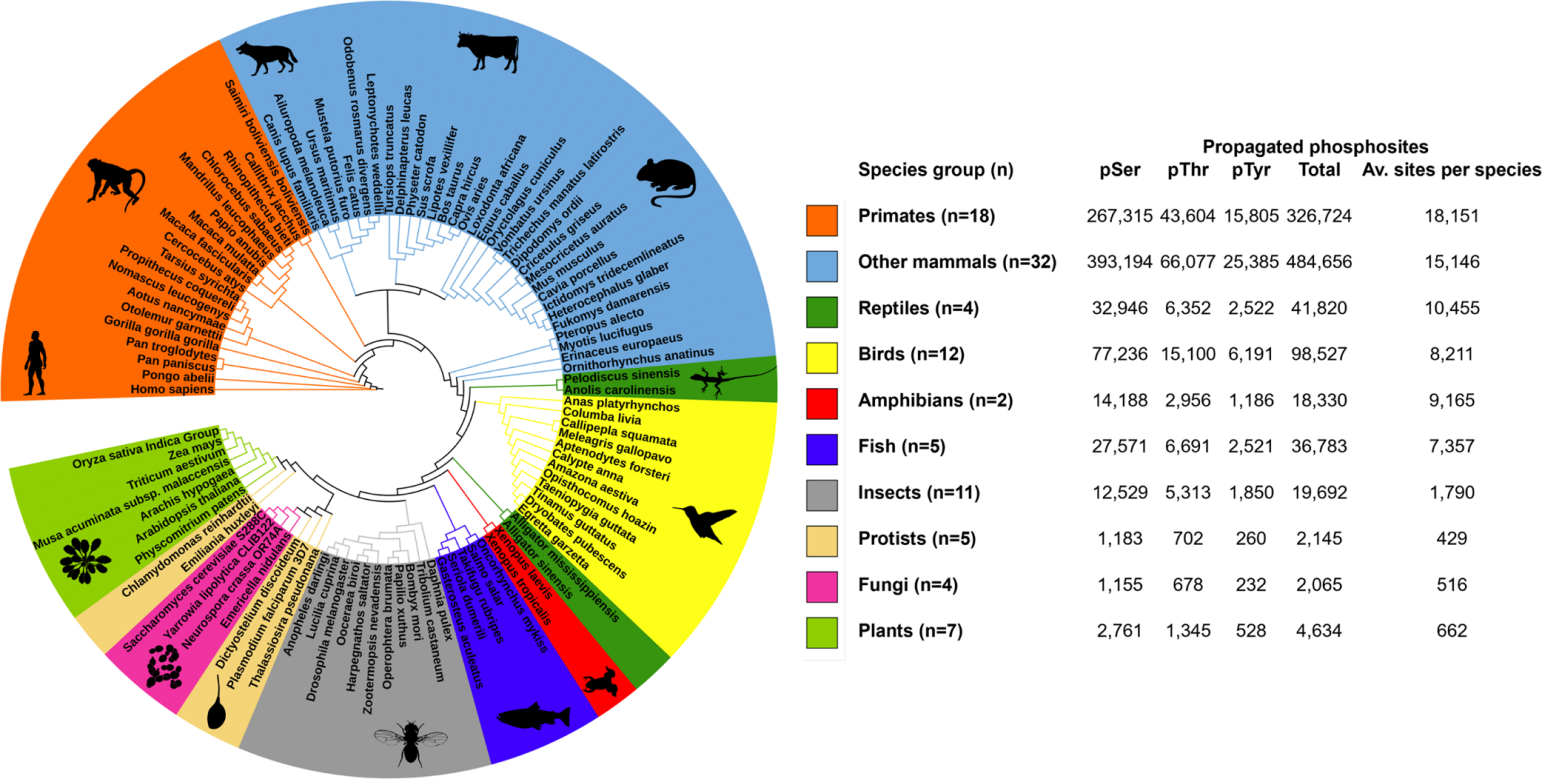
Phylogenetic relationship between the groups of eukaryotic species involved in the conservation analysis of human proteins and the resulting counts of propagated phosphosites from humans to other eukaryotes.

To validate our phosphosite predictions, we investigated how many of our predicted sites in species such as mouse and *Arabidopsis* had experimental identification evidence in PSP^18^ and Plant PTM Viewer,^21^ respectively. We found that 82% and 75% of our predicted Ser/Thr and Tyr phosphosites in mouse had reported experimental evidence in PSP, respectively (Table S4). Of those sites, 61% had at least 5 pieces of phosphorylation evidence (Table S4), indicating a confident set of phosphosites with a low false discovery rate. By considering the total number of Ser, Thr, and Tyr amino acids in the mouse proteome and the number of phosphosites reported in PSP for mouse, we estimated enrichment factors of 14 and 43 for identifying phosphorylation sites with any experimental evidence in PSP and those with at least 5 pieces of evidence, respectively, against a probability of identifying those sites by random chance in the mouse proteome. In other words, our method is much more likely to identify confident phosphosites in mice than if they were selected at random. Furthermore, we found that 35% of our predicted Ser, Thr, and Tyr phosphosites in *Arabidopsis* was supported by experimental evidence in Plant PTM Viewer resource (Table S4). The proportion of predicted *Arabidopsis* phosphosites mapped to experimental evidence was lower than that for the mouse predictions, likely because many of the target *Arabidopsis* protein sequences were missing phosphosite annotations or because many sequences have not yet been analyzed experimentally and reported in Plant PTM Viewer. Nevertheless, our results suggest that analyzing multiple sequence alignments between human proteins with confident phosphosites and their top sequence hits in BLAST from other species can successfully predict confident phosphosites in those species and thus act as a robust starting point for further analysis.

## Conclusions

A major strength of our approach lies in the use of established, freely available, high-quality human phosphorylation data to explore the functional importance of phosphosites. This allowed us to infer the relevance of phosphorylation in several ancient and novel protein functions, explore their specific roles, and analyze their associated protein domains. Our results emphasize the importance of conservation analysis in predicting functional significance of phosphosites and identifying organisms that can be used as models to study conserved signaling pathways relevant to human biology and disease.

To further understand the evolution of phosphorylation across eukaryotes, we analyzed the conservation of human kinases that are most likely responsible for phosphorylating “gold standard” phosphosites. As with phosphosites, we found various kinase conservation patterns across our selected eukaryotes. However, we could not find any significant correlation between the general conservation of phosphosites and their top matched kinases, likely due to the complexities of kinase-mediated phosphorylation signaling networks that were not considered in the current study. Nevertheless, our results provide a useful starting point for individual evolutionary investigations of target phosphosites and their respective kinases, particularly for the inference of signaling mechanisms between different eukaryotic species.

Finally, by using the “gold standard” human phosphosite set as a reference, we predicted over 1,000,000 potential high-confidence Ser, Thr, and Tyr phosphosites across various eukaryotic species ranging from primates and other mammals to plants, fungi, and protists. We envisage that researchers will use our “gold standard” human phosphosites (Table S1) and the predicted phosphosites in related eukaryotes (Table S4) as a basis for improving protein sequence annotations in different eukaryotic species and directing further research involving the use of those species as biological models for studying conserved cell signaling pathways in which phosphorylation plays an important role. The development of deep-learning tools such as AlphaFold3 ^99^ for predicting protein structures in the context of phosphorylation makes structural informatics one obvious setting for this to occur. Furthermore, our approach may be useful for characterizing the rapidly emerging links between ancestral prokaryotic Ser/Thr kinases and bacterial phosphoproteomes,^100^ as well as exploring phosphorylation of residues other than Ser, Thr, and Tyr.^101,102^

## Supplementary Material











## Data Availability

Supplementary file “alignments.zip”. Complete multiple sequence alignments in the aligned FASTA format between each human protein target and its top protein matches in BLAST from the selected eukaryotic species. Supplementary file “proteomes.zip”. Complete proteomes of analyzed eukaryotic species in the FASTA format can be accessed at 10.5281/zenodo.15005439.

## Notes

Top 10 functional categories for which protein sets containing target phosphosites with different conservation patterns significantly enriched in DAVID; conservation patterns of individual human phosphosites in selected groups of eukaryotic species; boxplots of human phosphosite conservation, also categorized by phosphosite localization in ordered or disordered protein regions; conservation patterns of mapped human Ser/Thr kinases within the groups of eukaryotic species; linear regression analysis comparing the conservation of Ser/Thr phosphosites with the conservation of their top matched kinase candidates; and boxplots comparing conservation between phosphosites involved in regulating protein–protein interactions and those that are not involved (PDF)
Complete data summary of “gold standard” human phosphosites (XLSX)
Summary of selected eukaryotic species (XLSX)
Summary of mapped human kinases and their orthologue data from OrthoDB (XLSX)
Summary of over 1,000,000 phosphosites propagated to eukaryotic species (XLSX)
