# Supplementary material for: Applying a Conservation-Based Approach for Predicting Novel Phosphorylation Sites in Eukaryotes and Evaluating Their Functional Relevance
Source: J Proteome Res. 2025 Jul 29;24(9):4547–62. doi: 10.1021/acs.jproteome.5c00278 (PMC12418505; doi:10.1021/acs.jproteome.5c00278)
Supplement: Supplementary file 1 [file pr5c00278_si_001.pdf]

Applying a conservation-based approach for predicting novel phosphorylation sites in eukaryotes and evaluating their functional relevance

Anton Kalyuzhnyy[1,2], Patrick A Eyers[1], Claire E Eyers[1,3], Eric W Deutsch[4], Andrew R Jones[1,2 *].

*Corresponding author email: Andrew.Jones@liverpool.ac.uk

1. Department of Biochemistry, Cell and Systems Biology, Institute of Systems, Molecular and Integrative Biology, University of Liverpool, Liverpool L69 7BE, UK.
2. Computational Biology Facility, Faculty of Health & Life Science, University of Liverpool, Liverpool L69 7BE, UK.
3. Centre for Proteome Research, Faculty of Health & Life Science, University of Liverpool, Liverpool L69 7BE, UK.
4. Institute for Systems Biology, Seattle, Washington 98109, United States.

Supplementary Information

File	Location
**Figure S1.** Top 10 functional categories for which protein sets containing target phosphosites with different conservation patterns were significantly enriched in DAVID	Page 2
**Figure S2.** Conservation patterns of individual human phosphosites in selected groups of eukaryotic species	Page 3
**Figure S3.** Boxplots of human phosphosite conservation, categorised by phosphosite localisation in ordered or disordered protein regions	Page 4
**Figure S4.** Conservation patterns of mapped human Ser/Thr kinases within the groups of eukaryotic species	Page 5
**Figure S5.** Linear regression analysis comparing the conservation of Ser/Thr phosphosites with the conservation of their top matched kinase candidates	Page 6
**Figure S6.** Boxplots of human phosphosite conservation, categorised by phosphosite involvement in regulating protein-protein interactions based on PTMint data	Page 7
**Table S1.** Table S1.xlsx. A complete data summary of “gold standard” human phosphosites	Submitted separately
**Table S2**. A summary of selected eukaryotic species	Submitted separately
**Table S3.** A summary of mapped human kinases and their orthologue data from OrthoDB	Submitted separately
**Table S4.** A summary of over 1,000,000 phosphosites propagated to eukaryotic species	Submitted separately
**File “*proteomes.zip*”.** Complete proteomes of analysed eukaryotic species in FASTA format	Access at: https://doi.org/10.5281/zenodo.15005439
**File “*alignments.zip*”.** Complete multiple sequence alignments in aligned FASTA format between each human protein target and its top protein matches in BLAST from the selected eukaryotic species	Access at: https://doi.org/10.5281/zenodo.15005439

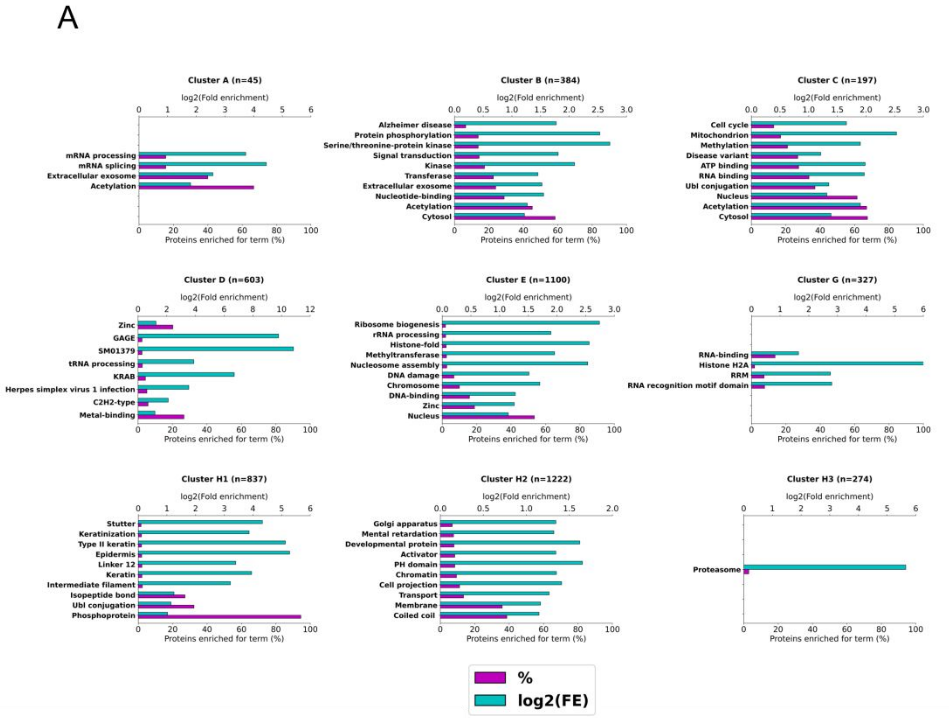

Cluster	Conservation pattern
A	High in all vertebrates except birds
B	High in animals only
C	High or Medium in all species
D	High in primates only
E	High in mammals only
G	High in mammals/Medium in reptiles
H1	High in all vertebrates except fish
H2	High in vertebrates only
H3	Medium in all vertebrates except amphibians

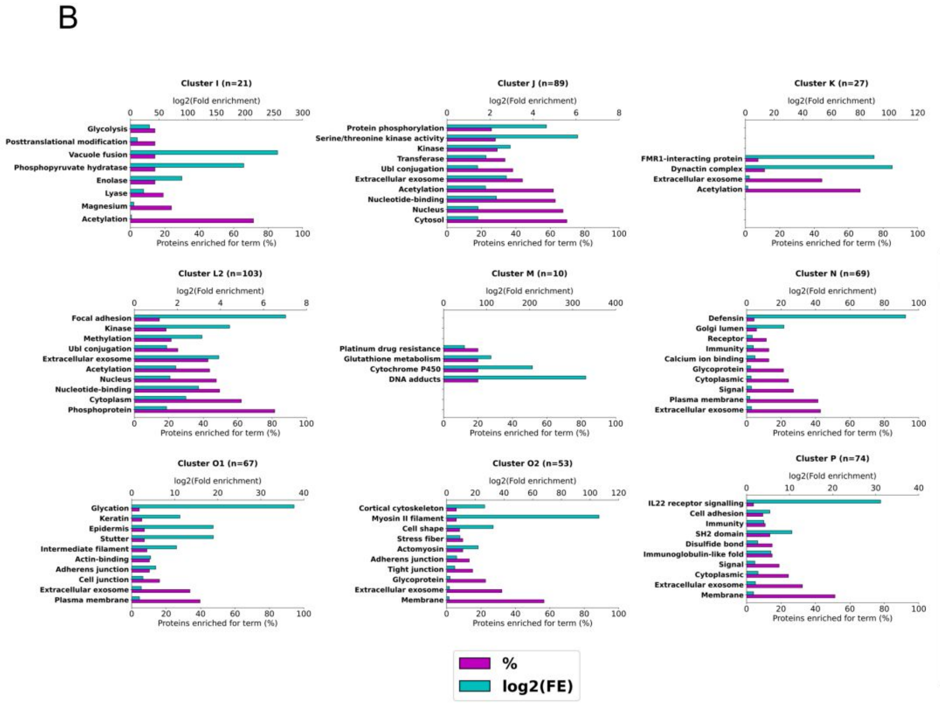

Cluster	Conservation pattern
I	High in all vertebrates/Medium in plants
J	High in all species
K	Medium in all species except fungi and protists
L1	High in vertebrates only
L2	High in animals only
M	Medium in primates only
N	High in mammals only
O1	Medium in all vertebrates except fish and amphibians
O2	High in all vertebrates except fish
P	Medium in all vertebrates except birds

Figure S1. Top 10 functional categories (where possible) for which protein sets containing **(A)** Ser/Thr and **(B)** Thr with different conservation patterns were significantly enriched in DAVID (Benjamini–Hochberg corrected p-value <0.1). For each protein set, the % of proteins enriched for a particular functional term is given as well as the log2(fold enrichment) for that set. The number of proteins in each set is presented by n.

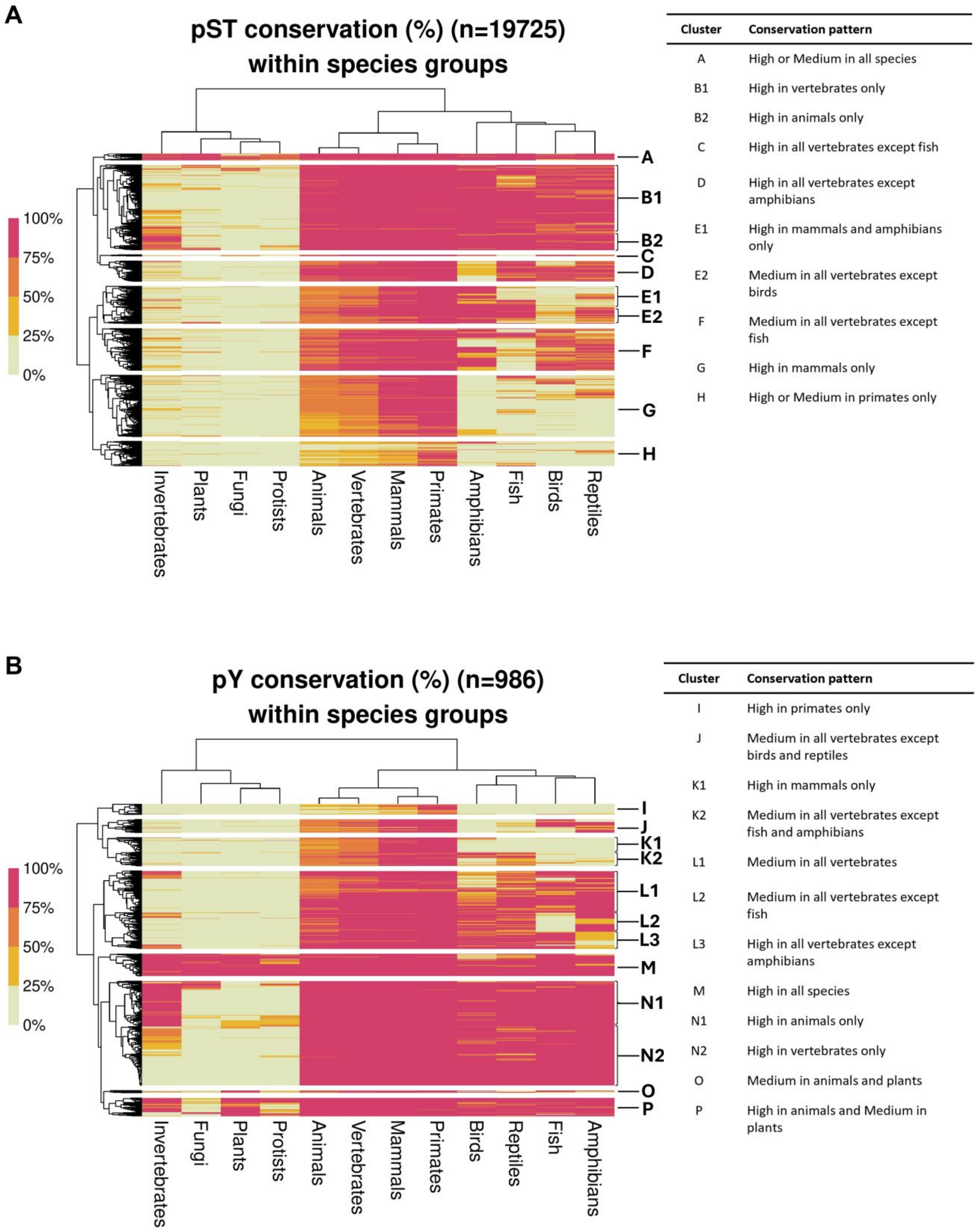

Cluster	Conservation pattern
A	High or Medium in all species
B1	High in vertebrates only
B2	High in animals only
C	High in all vertebrates except fish
D	High in all vertebrates except amphibians
E1	High in mammals and amphibians only
E2	Medium in all vertebrates except birds
F	Medium in all vertebrates except fish
G	High in mammals only
H	High or Medium in primates only

Cluster	Conservation pattern
I	High in primates only
J	Medium in all vertebrates except birds and reptiles
K1	High in mammals only
K2	Medium in all vertebrates except fish and amphibians
L1	Medium in all vertebrates
L2	Medium in all vertebrates except fish
L3	High in all vertebrates except amphibians
M	High in all species
N1	High in animals only
N2	High in vertebrates only
O	Medium in animals and plants
P	High in animals and Medium in plants

Figure S2. Conservation patterns of **(A)** Ser/Thr and **(B)** Tyr phosphosites from human proteins across the groups of eukaryotic species. Each row in the heatmap represents an individual phosphosite and its conservation across specific species groups which are separated into columns. Conservation is scored as a percentage out of all species per group and reflected by a colour gradient divided at quarterly intervals. Phosphosites were clustered based on their similarity in conservation patterns using the Euclidean distance method. For each cluster, a label is assigned which describes the most observed conservation pattern (i.e., at least 50% of sites in the cluster follow the described phosphosite conservation pattern), where high and medium conservation refers to conservation scores of ≥75% and ≥50%, respectively. The total number of analysed phosphosites is given by n.

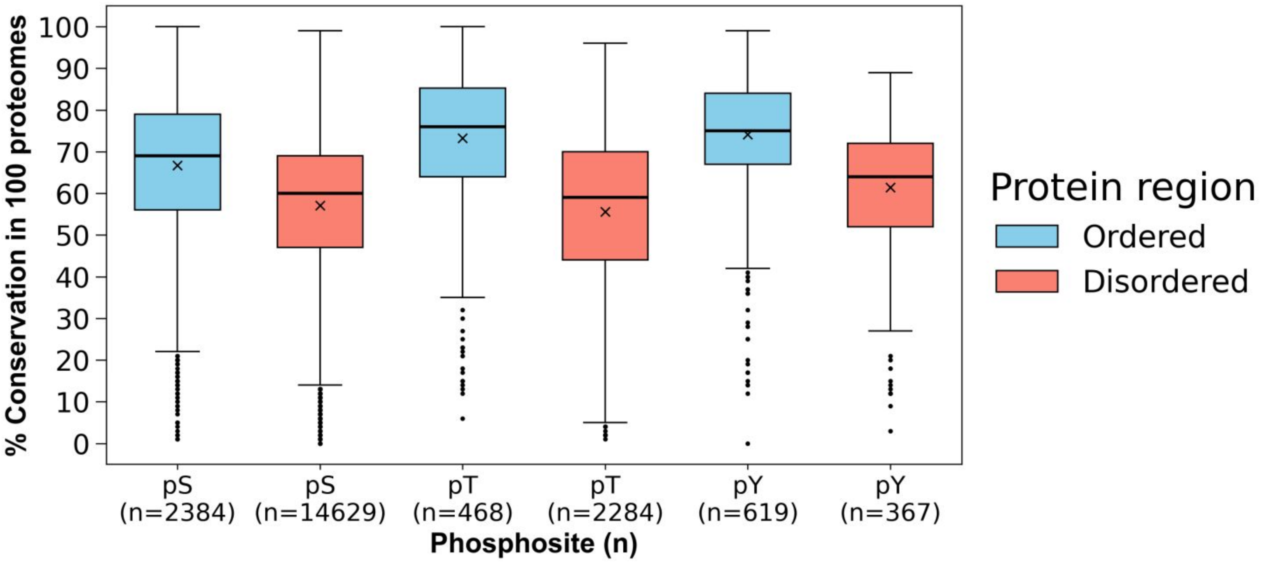

Figure S3. Boxplots of conservation percentages (%) across 100 eukaryotic species of human Ser, Thr and Tyr phosphosites categorised by whether they are found in ordered or disordered protein regions. As per Tukey's boxplot definition, each box extends from the first quartile to the third quartile of the data, with a line at the median % conservation. The whiskers extend from the box to the farthest data point lying within 1.5x the interquartile range from the box. An (x) symbol represents mean % conservation per group and dots (•) represent outlier values.

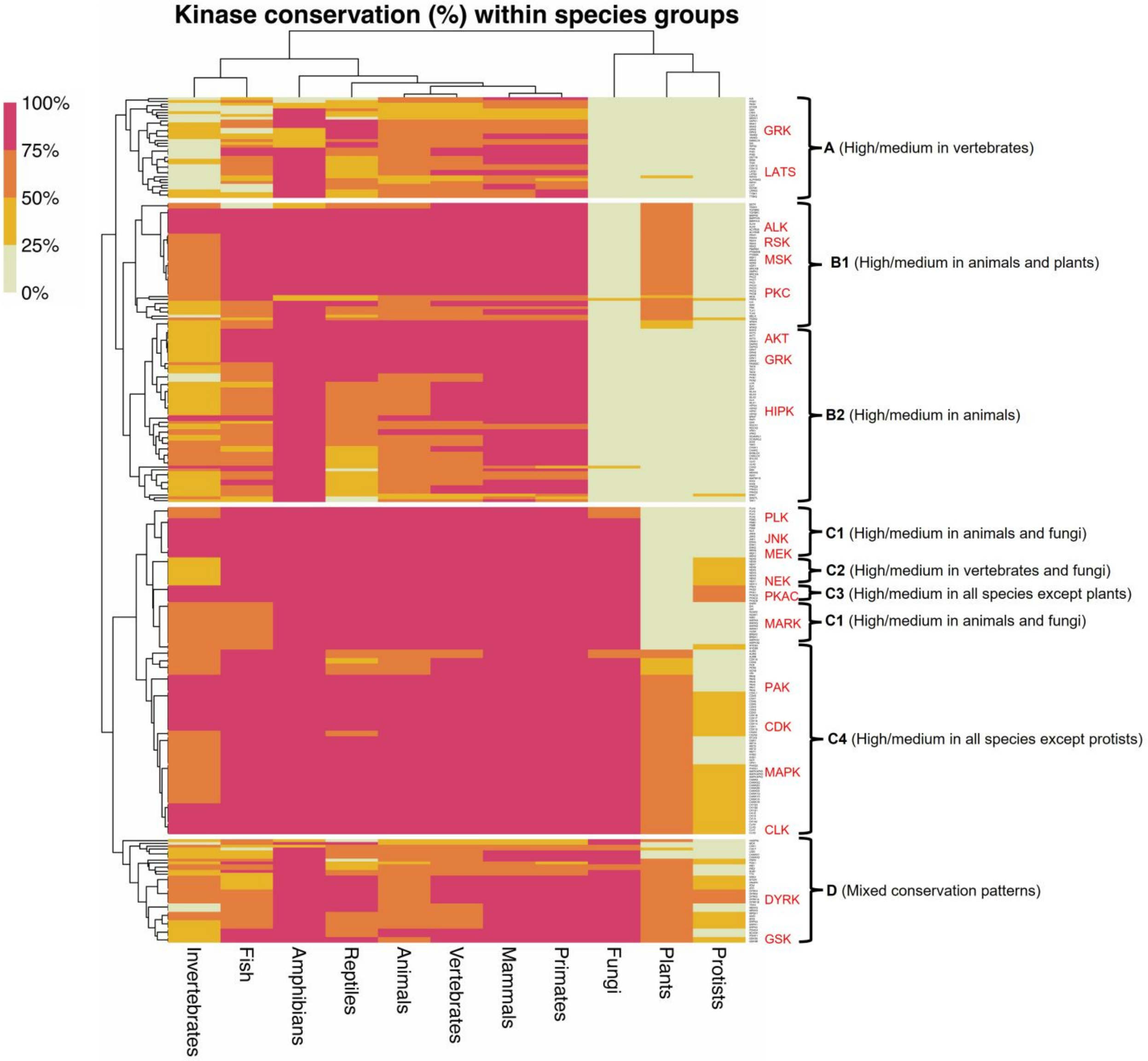

Figure S4. Conservation patterns of human Ser/Thr kinases within the groups of eukaryotic species. Each row in the heatmap represents an individual kinase and its conservation across specific species groups which are separated into columns. Conservation is scored as a percentage out of all species per group and reflected by a colour gradient divided at quarterly intervals. The kinases are clustered based on their similarity in conservation patterns using the Euclidean distance method. For each cluster, a label is assigned which describes the most observed conservation pattern (i.e., at least 50% of kinases in the cluster follow the described phosphosite conservation pattern), where high and medium conservation refers to conservation scores of ≥75% and ≥50%, respectively. The total number of analysed kinases is given by n.

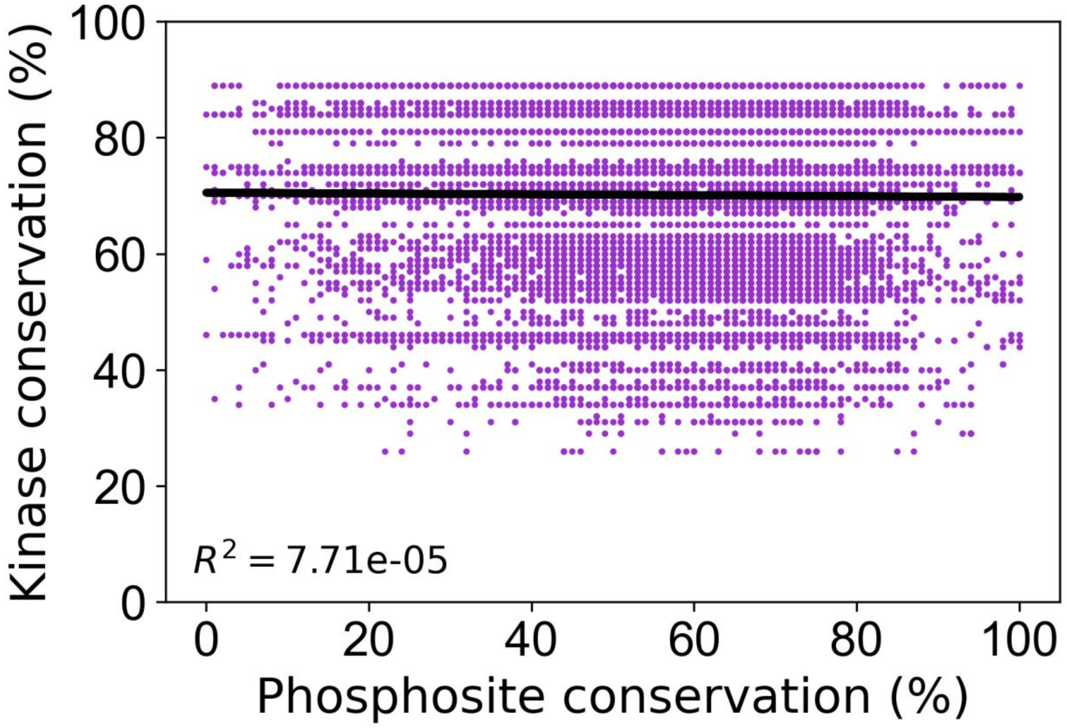

Figure S5. Linear regression analysis comparing the conservation of Ser/Thr phosphosites in 100 eukaryotic species with the conservation of their top matched kinase candidates. The R^2 coefficient demonstrates that there is effectively no linear relationship between phosphosite and kinase conservation. The black solid line represents the line of best fit.

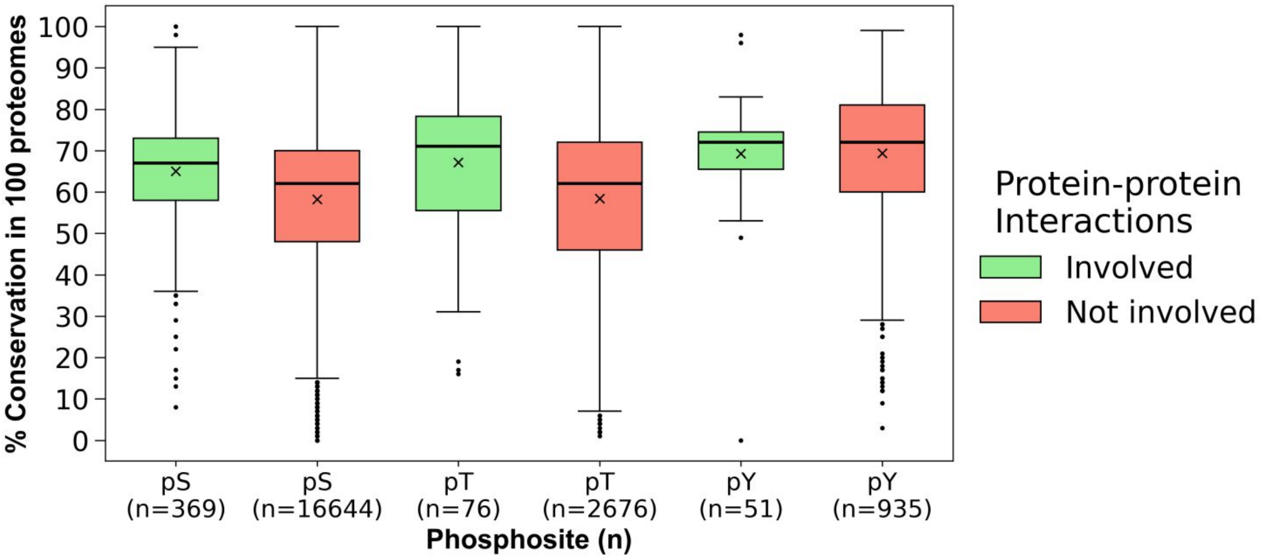

Figure S6. Boxplots of conservation percentages (%) across 100 eukaryotic species of human Ser, Thr and Tyr phosphosites categorised by whether they are involved in regulating protein-protein interactions based on the data from the PTMint database. As per Tukey's boxplot definition, each box extends from the first quartile to the third quartile of the data, with a line at the median % conservation. The whiskers extend from the box to the farthest data point lying within 1.5x the interquartile range from the box. An (x) symbol represents mean % conservation per group and dots (•) represent outlier values.
